# Genome-Wide Survey of Leucine-Rich Repeat Receptor-Like Protein Kinase Genes and CRISPR/Cas9-Targeted Mutagenesis *BnBRI1* in *Brassica napus*

**DOI:** 10.3389/fpls.2022.865132

**Published:** 2022-04-12

**Authors:** Min Song, Bin Linghu, Shuhua Huang, Fang Li, Ran An, Changgen Xie, Yantao Zhu, Shengwu Hu, Jianxin Mu, Yanfeng Zhang

**Affiliations:** ^1^Hybrid Rapeseed Research Center of Shaanxi Province, Yangling, China; ^2^State Key Laboratory of Crop Stress Biology in Arid Areas and College of Agronomy, Northwest A&F University, Yangling, China

**Keywords:** *Brassica napus*, LRR-RLK, CRISPR/Cas9, *BnBRI1*, semi-dwarf

## Abstract

The leucine-rich repeat receptor-like protein kinase (*LRR-RLK*) family represents the largest group of RLKs in plants and plays vital roles in plant growth, development and the responses to environmental stress. Although *LRR-RLK* families have been identified in many species, they have not yet been reported in *B. napus*. In this study, a total of 444 *BnLRR-RLK* genes were identified in the genome of *Brassica napus* cultivar “Zhongshuang 11” (ZS11), and classified into 22 subfamilies based on phylogenetic relationships and genome-wide analyses. Conserved motifs and gene structures were shared within but not between subfamilies. The 444 *BnLRR-RLK* genes were asymmetrically distributed on 19 chromosomes and exhibited specific expression profiles in different tissues and in response to stress. We identified six *BnBRI1* homologs and obtained partial knockouts via CRISPR/Cas9 technology, generating semi-dwarf lines without decreased yield compared with controls. This study provides comprehensive insight of the *LRR-RLK* family in *B. napus*. Additionally, the semi-dwarf lines expand the “ideotype” germplasm resources and accelerate the breeding process for *B. napus*.

## Introduction

Receptor-like protein kinases (RLKs) serve as receptors for signaling transduction pathways that regulate many biological process (Clark et al., 1997; Li, 2010; Xun et al., 2020; Lu et al., 2021). Leucine-rich repeat receptor-like protein kinases (LRR-RLKs) comprise one of the largest groups of RLKs (Liu et al., 2017). There are 200–300 LRR-RLKs in *Arabidopsis*, tomato, rice, potato, and maize, respectively (Song et al., 2015; Wei et al., 2015; Bettembourg et al., 2017; Sun J. et al., 2017; Li et al., 2018). LRR-RLKs usually contain an extracellular, tandemly organized LRR domain (20–30 amino acid residues), a single-pass transmembrane domain, and functional protein kinase domain (Mishra et al., 2021).

LRR-RLKs are highly conserved, widely distributed and play vital roles in plant growth, development and stress responses. For instance, SERK3/BAK1 function in the plant immunity, growth, and cell death (Zhou et al., 2019). *HSL3* function in regulating plant stomatal closure and the drought stress response through modulate hydrogen peroxide homeostasis (Liu et al., 2020). AtPXL1 functions in signal transduction pathways that respond to temperature fluctuations (Jung et al., 2015). *XIP1/CEPR1* and *CEPR2* are involved in the regulation of lateral root growth in *Arabidopsis* (Dimitrov and Tax, 2018). *OsDOCS1* plays critical roles in plant root cap development (Bettembourg et al., 2017). *OsSTLK* regulates salt stress tolerance, potentially by regulating the ROS scavenging system, Na^+^/K^+^ ratio and MAPK signaling pathway (Lin et al., 2020).

The LRR-RLK BRI1 encodes a receptor serine/threonine kinase and has an extracellular domain that contains 25 leucine-rich repeats. BRI1 and BAK1 interact and regulate brassinosteroid signaling in *Arabidopsis*, and BRI1 can phosphorylate BAK1. *Arabidopsis* overexpressing BRI1 are highly sensitive to brassinosteroid and have higher brassinosteroid binding activity (Nam and Li, 2002). BRI1 is an important plasma-membrane receptor for plant steroids, as shown by immunoblotting and brassinolide (BL)-induced BRI1 phosphorylation (Wang et al., 2001). In wheat, knockdown of *TaBRI1* reduces photosynthesis, the responses to light and temperature stresses, and yield (Fang et al., 2020). In potato, knockdown of *BRI1* attenuates brassinosteroid signaling and decreases plant height (Huang et al., 2021). In *Brachypodium distachyon*, down-regulation of *BdBRI1* expression results in reduced plant height, shortened internodes, as well as narrow and short leaves (Feng et al., 2015). In rice, loss-of-function of *OsBRI1* reduced internode elongation and bending of the lamina joint (Yamamuro et al., 2000).

The rapeseed (*Brassica napus* L., AACC, 2n = 4x = 38) originated from the spontaneous hybridization of *B. oleracea* and *B. rapa*. It is the second largest oilseed crop in the world, and plays a crucial role in the production of edible oil (Zhu et al., 2016). Plant height and branch angle are key factors of rapeseed architecture, which is a major determinant of plant yield (Fan et al., 2021; Stanic et al., 2021). Taller rapeseed is not only unfavorable for mechanized harvesting, but also reduces the overall yield. We propose that an “ideotype” for rapeseed includes a semi-dwarf and compact architecture (Fu et al., 2016).

CRISPR/Cas9-mediated genome editing technology creates the opportunity to establish powerful germplasm resources (Zhang et al., 2021). However, there are few reports on germplasm resources of optimal plant-type, and no systematic study of LRR-RLK family in *B. napus*. In our study, we identified *LRR-RLK* genes in the rapeseed genome, and performed a systematic analysis of their phylogenetic relationship, chromosomal locations, gene structure, conserved protein domains, tandem and segmental duplication events, collinearity, and expression profiles. In addition, we used a CRISPR-Cas9 strategy to knockout *BnBRI1* homologues to create ideotype mutants with semi-dwarf structure, good yield, and increased breeding potential.

## Materials and Methods

### Identification of Leucine-Rich Repeat Receptor-Like Protein Kinases in *Brassica napus*

A total of 225 AtLRR-RLKs protein sequences were download from the TAIR database^1^ (Sun J. et al., 2017). Protein sequences of the semi-winter rapeseed cultivar ‘‘Zhongshuang11’’ (ZS11) were download from the National Genomics Data Center (NGDC),^2^ and the accession number was PRJCA002883. The longest transcript of each gene was used for subsequent identification. Hidden Markov Model (HMM) and BLASTP search were used to classify the BnLRR-RLKs. The HMM profiles of LRR domain LRR1 (PF00560), LRR3 (PF07725), LRR4 (PF12799), LRR5 (PF13306), LRR6 (PF13516), LRR8 (PF13855), LRR9 (PF14580), LRR10 (PF18805), LRR11 (PF18831), LRR12 (PF18837), LRRNT-2 (PF08263), and Pkinase domain (PF00069) as well asPkinase_Tyr (PF07714) were obtained from the Pfam database.^3^

HUMMER3.1 software of Linux version was used to take the intersection of LRR and Pkinase domain, and the threshold was *E*-value < e^–10^. The 225 AtLRR-RLK protein sequences were used as queries to perform a BLASTP search in the local protein database of ZS11 with an *E*-value < e^–10^ and identity ≥ 50% (Camacho et al., 2009), and the putative BnLRR-RLKs were obtained by taking the intersection of the HUMMER and Blastap methods. Finally, these BnLRR-RLKs were submitted to the NCBI-CDD server^4^ and the SMART database^5^ to screen for proteins with the LRR and Pkinase domains. In addition, the theoretical isoelectric points (pI) and molecular weight (MW) of the LRR-RLKs were calculated by using the ExPASy database.^6^

### Phylogenetic Trees, Conserved Motif, and Gene Structure Analysis BnLRR-RLKs

Multiple sequence alignment of the full-length BnLRR-RLK proteins were performed with the FFT-NS-I method of the MAFFT software (Katoh et al., 2019). Fasttree software was used as the maximum likelihood method for constructing phylogenetic trees (Price et al., 2009). The EvolView software was used to visualize the phylogenetic tree (Zhang et al., 2012). Conserved motifs (the maximum is set to 10) were predicted using the MEME v5.2.0 with the maximum number of motifs and the optimum width of each motif falls between 10 and 200 residues (Bailey et al., 2009). The structures of the BnLRR-RLK genes were displayed based on GFF annotation files by TBtools software (Chen et al., 2020).

### Chromosomal Location, Gene Duplication, and Genomic Synteny of *BnLRR-RLKs*

Chromosome location information of the *BnLRR-RLK* genes was extracted from the GFF file, and plotted by the MapChart version 3.0 software (Voorrips, 2002). In order to accurately analyze *BnLRR-RLK* duplication events, the e-value of e^–100^ was used to align all of the protein sequences in ZS11 with the BLASTP program (Camacho et al., 2009). The duplication pattern of these genes were detected with the default parameters of the MCScanX software, and divided into tandem duplication and segmental duplication (Krzywinski et al., 2009). Similarly, the e-value of e^–100^ was used to align all of the protein sequences between ZS11 and *Arabidopsis* with the BLASTP program, and all syntenic blocks were mapped with JCVI software (Tang et al., 2008).

### Expression Patterns of *BnLRR-RLKs*

RNA-seq data from 12 tissues (root, stem, leaf, flower, silique, sepal, pistil, stamen, ovule, pericarp, wilting pistil, and blossomy pistil) of ZS11 were downloaded from the NCBI database (project ID: PRJNA394926) (Sun F. M. et al., 2017). RNA-seq data of ZS11 leaf under abiotic stress, including dehydration, salt, ABA and cold stress treatments, were downloaded from the NGDC database (project ID: CRA001775) (Zhang et al., 2019). These transcriptome data were mapped to the ZS11 reference genome using HISAT2 software (Kim et al., 2015). The TPM values (Transcripts Per Million) were calculated by FeatureCounts R package and the heatmaps were presented using TBtools software (Liao et al., 2014; Chen et al., 2018).

### CRISPR/Cas9 Vector Construction and Transformation

To construct the CRISPR/Cas9 recombinant plasmid, a common target sequence of six *BnBri1* genes was designed (Supplementary Table 1), and this target dsDNA was generated via direct annealing of two oligonucleotides primers, and assembled into the pHSE401 vector (Xing et al., 2014). The resulting pHSE401-BRI1 constructs were transformed into the agrobacterium tumefaciens strain GV3101 by electroporation and subsequently transformed into the selfing line K407 of *B. napus* using the method described previously (Bhalla and Singh, 2008).

Genomic DNA extracted from T_3_ plants was used for PCR amplification with genotyping primers (Supplementary Table 1), the PCR products were conducted by sanger sequencing, and cloned into a pEASY-Blunt cloning vector (TransGen Biotech, Beijing, China), positive single clones were sequenced to identify mutations (Aoke Biotech, Beijing, China).

### Analysis of Seedlings Hypocotyl Length and Growth Response to Brassinozole

To analyze the hypocotyl variation of the gene-edited mutants, the seeds were sown in humus soil under 16 h light/8 h dark photoperiod at 22^°^C, and the hypocotyl length was measured after 6 days. To verify the response to Brassinozole (BRZ), the seeds of mutants were sterilized (75% alcohol for 30 s, 15% NaClO for 8 min, rinse 5 times with sterile water) and bunch planting at 1/2 strength Murashige and Skoog solid medium (1/2 MS), which contained 0 and 1,000 nM BRZ, respectively (Tokyo Chemical Industry Co., Ltd., Tokyo, Japan). After 5 days of darkness cultivation at 22^°^C, the hypocotyl length was measured. Each treatment was repeated three times.

### Quantitative Real-Time PCR Analysis

To detect the expression level of *BnBRI1*, total RNA was extracted from seedlings of the L18, L24 and WT by RNAiso Plus kit (TaKaRa, Dalian, China). One microgram of RNA was reverse-transcribed to cDNA using One-Step gDNA Removal and cDNA Synthesis SuperMix (TransGen Biotech, Beijing, China). qRT-PCR was performed using a SYBR Green Master Mix kit (TaKaRa, Dalian, China). The *BnUBC21* gene was used as an internal reference, the primer pairs are listed in Supplementary Table 1. Each sample contains 3 biological replicates and 3 technical replicates.

### Statistical Analysis

All data were analyzed with One-way analysis of variance (ANOVA) of SPSS 26 software. GraphPad prism (version 8.0.2) was used for drawing graphs. Each experiment was repeated at least three times, and significant differences are indicated at the significance levels of *P* ≤ 0.05.

## Results

### Identification of Leucine-Rich Repeat Receptor-Like Protein Kinase Gene Families in *Brassica napus*

We detected a total of 444 *BnLRR-RLK* genes in the genome of *B. napus* cultivar ZS11, which is nearly double the number of *LRR-RLK* genes in *Arabidopsis* (Table 1). Specifically, we uncovered 215 *LRR-RLK* genes in the A subgenome and 225 *LRR-RLK* genes in the C subgenome. We renamed all of the *BnLRR-RLK* genes on the basis of chromosomal localization. Gene position and other details are presented in Supplementary Table 2. We found that BnLRR-RLK protein lengths were between 459 and 1,303 amino acids (aa). In addition, the protein molecular weights (MV) ranged from 51.31 to 143.19 kDa, and the isoelectric point (pI) from 4.77 to 10.27.

**TABLE 1:** Gene numbers of identified *LRR-RLK* subfamily in *B. napus* and *A. thaliana.*

LRR-RLK subfamily	*A. thaliana*	*B. napus*	
		A	C
I-1	48	19	21
I-2	2	0	0
II	14	16	14
III	46	50	49
IV	3	4	4
IX	4	7	7
V	9	9	9
VI-1	5	6	4
VI-2	8	5	6
VII-1	5	4	6
VII-2	3	3	3
VII-3	2	2	2
VIII-1	8	3	6
Xa	4	5	5
Xb-1	9	12	14
Xb-2	1	1	1
XI-1	33	40	43
XI-2	2	2	3
XII-1	8	17	21
XIIIa	4	3	4
XIIIb	3	3	3
XIV	2	3	3
XV	2	1	1
Total numbers	225	215	229
		444	

### Phylogenetic Relationship, Conserved Motif, and Gene Structure Analysis

**FIGURE 1:**
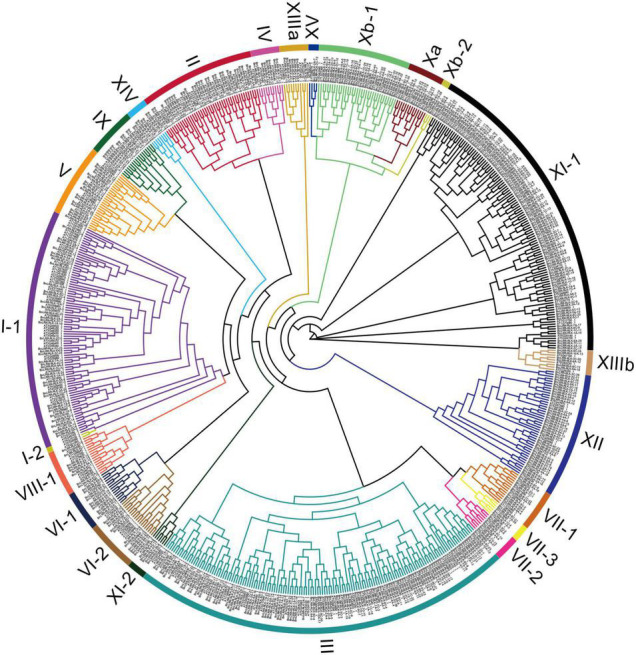
Phylogenetic tree analysis of *BnLRR-RLK* gene family in *B. napus*. Fasttree software was used as the maximum likelihood method for constructing phylogenetic trees with the protein sequences of *B. napus* and *A. thaliana*. The color clade illustrates the different subfamilies.

To evaluate the evolutionary relationships between *LRR-RLK* genes in *B. napus* and *A. thaliana*, we constructed a phylogenetic tree using the 444 BnLRR-RLK full-length proteins together with 225 AtLRR-RLK proteins. The 444 *BnLRR-RLKs* were subdivided into 22 groups (I–XV) (Figure 1). The largest group, LRR-RLK-III, contained 99 genes. In contrast with *A. thaliana*, no genes were identified in group I-2. There were at least twice as many *LRR-RLK* genes in each subgroup of *B. napus* vs. *A. thaliana*, except for groups I-1, VI-2, VIII-1, and XV, which had a similar number of *LRR-RLK* genes in *B. napus* and *A. thaliana*. These data suggest that allopolyploidization contributed to the expansion of *LRR-RLKs* in *B. napus*, but that not every subfamily has an increased the number of genes.

In addition, we analyzed conserved motifs and gene structures to define *BnLRR-RLK* family characteristics. Using MEME tools, we identified 10 putative motifs (Supplementary Table 3). We found that the type, number, and arrangement of the N-terminus determined the classification of different subfamilies, and expanded the variety and quantity of *LRR-RLKs* (Supplementary Figure 1). The N-termini of these proteins were enriched for many different tandem repeat motifs, such as motif 1, motif 3 and motif 4. Intriguingly, motif 2 and motif 5 were often found at the C-terminus.

The gene structure characteristics revealed that different subfamilies shared different intron-exon distribution, whereas members within the same subfamily had a similar gene structure, suggesting a similar evolution within subgroups but not between subgroups, based on GFF annotation files (Supplementary Figure 1). The large number of intron-exons in subgroups I-1, II, XIII, V, and VI-2, indicate a complex gene structure, whereas other subfamilies contained few intron-exons and showed simpler gene structures.

### The Distribution, Gene Duplication, and Genomic Synteny

Knowledge of the chromosome distribution of gene family members is essential for studying family member duplication and collinearity. We found that the 444 *BnLRR-RLK* genes were widely distributed on 19 chromosomes, and the distribution was extremely uneven, with about 40 genes on chromosome 3C and 13 genes on chromosomes 4A and 8A, respectively (Supplementary Figure 2). We did not observe any preference for the distribution of *LRR-RLK* genes on each chromosome, with the exception that there were no *LRR-RLK* genes on the upper arm of chromosome 4A.

**FIGURE 2:**
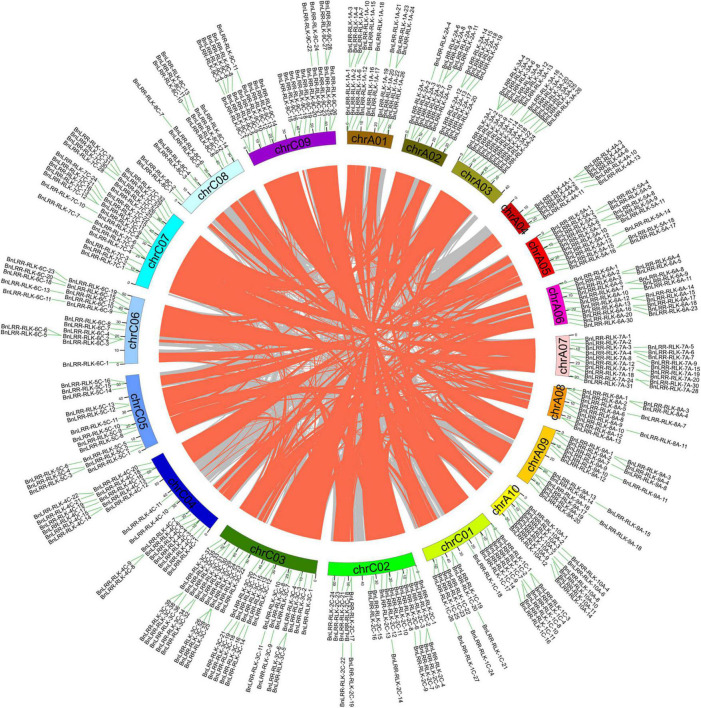
The chromosome locations associated with *BnLRR-RLKs* segmental duplication in *B. napus*. The gray and orange lines represent all synteny blocks in *B. napus* Cultivar ZS11 genome and segmental duplicate gene pairs, respectively. Different chromosomes are indicated by different colors.

Gene duplication is a major mechanism underlying the expansion of gene family members. Based on BLAST and MCScanX software, we identified a total of 6 tandem duplication events and 395 segmental duplications in the ZS11 genome (Figure 2 and Supplementary Tables 4, 5). Only 9 events were detected within the same chromosome, whereas 386 segmental duplications occurred across chromosomes, which suggests that segmental duplication events across chromosomes plays a key role in *LRR-RLKs* expansion. Further analysis showed that 51 duplication events occurred on the AA subgenome, 55 events on the CC subgenome, whereas 289 events occurred across AA/CC subgenomes. Considering that *B. napus* (AACC genome) derived from a spontaneous hybridization between *B. rapa* (AA genome) and *B. oleracea* (CC genome), and contains the complete diploid chromosome sets of both parental genomes. Therefore, we infer that allopolyploidization plays a significant role in the expansion of the *LRR-RLKs* in *B. napus*.

To investigate the evolution of the *LRR-RLKs*, we analyzed their synteny between *B. napus* and *A. Thaliana* at the whole genome level. A total of 374 gene pairs were detected between the two genomes (Supplementary Figure 3 and Supplementary Table 6). Most *AtLRR-RLK* genes have multiple orthologous genes in *B. napus*. For instance, *AtBRI1* (*AT4G39400*) corresponds to 6 *BnBRI1* genes, indicating that *BnBRI1* genes expanded in rapeseed. However, the 29 *AtLRR-RLKs* have only one collinear *BnLRR-RLK* gene.

### Expression Profiles of *BnLRR-RLKs* in Different Tissues

**FIGURE 3:**
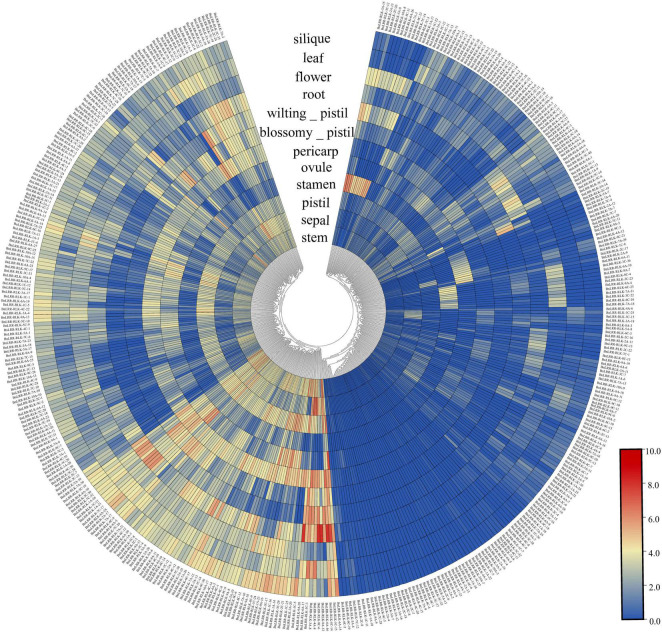
Expression profiles of *BnLRR-RLKs* in different tissues. Publicly available RNA-seq data of root, stem, leaf, flower, silique, sepal, pistil, stamen, ovule, pericarp, blossomy pistil, and wilting pistil from ZS11 were download for expression profile analysis. The expression level is equal to the mean values and transforms log2 values for normalization. Colors from blue to red represent relative expression levels from low to high.

The specific expression of genes in different tissues sheds light on gene function. In order to explore the function of the *BnLRR-RLKs*, we investigated their expression profiles in 12 different tissues, using public available RNA-seq data. We detected the expression of 426 *BnLRR-RLKs* in at least one tissue, whereas 18 genes were not expressed in any of the tissues analyzed (Figure 3 and Supplementary Table 7). We divided the 426 genes into two groups: group 1 displayed high expression levels in different tissues, whereas group 2 displayed low expression in tissues. For instance, *BnLRR-RLK-6A-15* was highly expressed in ovule, *BnLRR-RLK-8A-12* and *BnLRR-RLK-8C-6* were highly expressed in root. These results reflected that the expression of *LRR-RLKs* vary in different tissues, suggesting that *BnLRR-RLKs* play functionally diverse roles in tissue development.

### Expression Profiles of *BnLRR-RLKs* in Response to Abiotic Stresses

**FIGURE 4:**
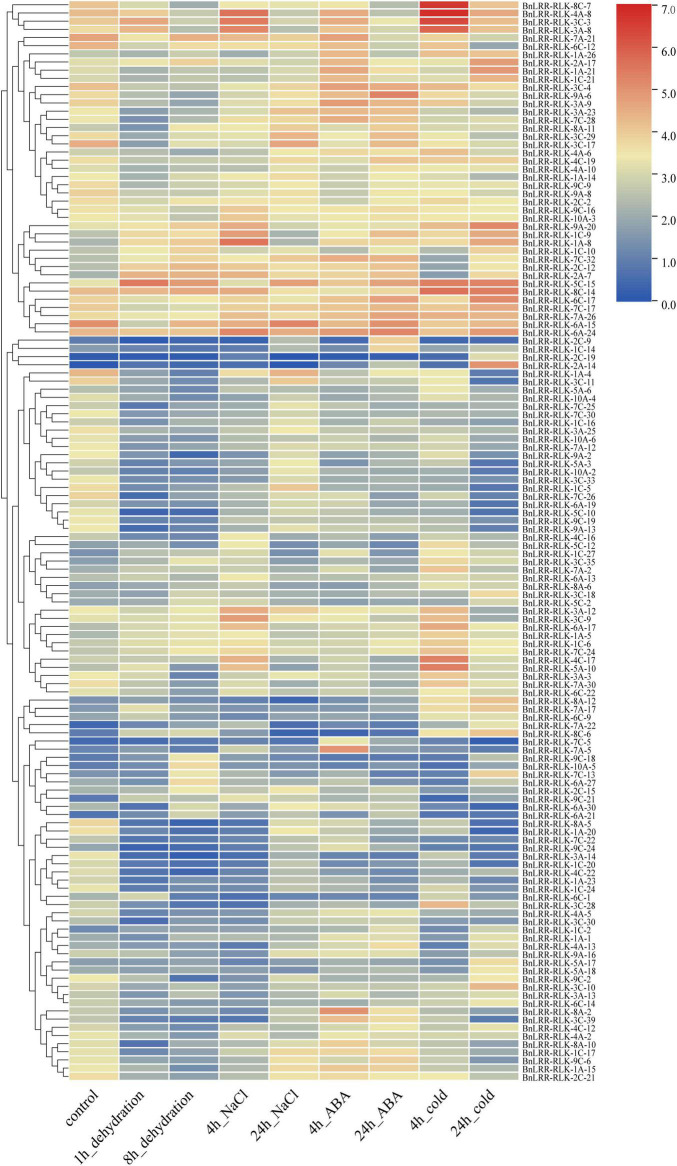
Expression profiles of *BnLRR-RLKs* under different stresses. Leaf RNA-seq data for ZS11 under abiotic stress, namely dehydration, salt, ABA and cold stress treatments, was downloaded from the NGDC database. Expression level is equal to the mean values and transforms log2 values for normalization. Colors from blue to red represent relative expression levels from low to high.

*LRR-RLK* genes play a major role in abiotic stress responses. To explore the expression of *LRR-RLK* genes in response to abiotic stress, we examined their expression patterns under four abiotic stresses (dehydration, salt, abscisic acid (ABA), cold) using the published transcriptome data of *B. napus*. A total of 132 genes were extracted from the expression matrix based on STDEV value > 2 among different treatments (Figure 4 and Supplementary Table 8). These genes clustered into two groups.

Group 1 contained 41 genes with significantly different expression patterns under different stress treatments. For example, *BnLRR-RLK-4A-8*, *BnLRR-RLK-3C-3*, and *BnLRR-RLK-3A-8* were all up-regulated after 4 h-salt or 4 h-cold treatment, suggesting they may be involved in the related stress response. Interestingly, some genes displayed opposing expression profiles under different treatments. For instance, *BnLRR-RLK-9A-6* was sharply down-regulated after 1 h-dehydration, but up-regulated after 4 h-ABA treatment. These data suggest that the responses to dehydration and ABA stress have opposing molecular mechanisms.

Group 2 contained 91 genes with low expression levels under each stress treatment. Unusually, part of the genes exhibited sharply changes in expression levels after a specific stress treatment, for instance, BnLRR-*RLK-4C-17* and *BnLRR-RLK-5A-10* were significantly up-regulated after 4 h-cold treatment, *BnLRR-RLK-7A-5* and *BnLRR-RLK-8A-2* were up-regulated after 4 h-ABA treatment, whereas *BnLRR-RLK-1A-4* and *BnLRR-RLK-3C-11* were strikingly down-regulated under dehydration treatment. The results provided useful information for studying the function of *LRR-RLKs* in response to abiotic stress.

### Knockout of *BnBRI1* Homologs by CRISPR/Cas9 Targeted Mutagenesis

**FIGURE 5:**
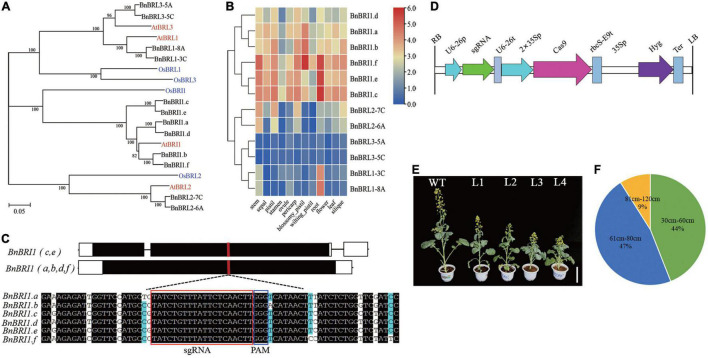
Evolutionary analysis of *BRI1* and *BRI1-like* receptor family and CRISPR/cas9 targeted *BnBRI1* gene mutation. **(A)** Phylogenetic tree analysis of BRI1-like receptor family in *B. napus* and *A. thaliana*. **(B)** Expression profiles of *BnBRI1* and *BnBRL* genes in different tissues. **(C)** CRISPR/Cas9 sgRNA targets the exon of *BnBRI1* genes. The red box highlights the target sequences in six *BnBRI1* homologous sequences. The protospacer adjacent motif (PAM) is indicated in the blue box. **(D)** Physical map of CRISPR/cas9 vector pHSE401 between the RB and LB. **(E)** Phenotypes of CRISPR/Cas9-edited *BnBRI1* mutants during flowering of rapeseed. From left to right are WT (K407), L1∼L4 strains, scale bars = 12 cm. **(F)** Pie chart of plant height from the gene edited *Bnbri1* mutants in T_0_ generation. The green, blue, and yellow indicates the percentage of extreme dwarf, dwarf, and semi-dwarf phenotypes, respectively.

Based on previous research in *Arabidopsis* and rice, we identified six *BRI1* orthologs (renamed as *BnBRI1.a*∼*BnBRI1.f*), and six *BRI1-Like* (*BRL*) genes in *B. napus* (Figure 5A and Supplementary Table 9). The *BnBRI1* and *BnBRL* genes both belonged to the Xb-1 subfamily, while the *BnBRI1.b* and the *BnBRI1.f* had higher homology with *AtBRI1*. Clustering analysis based on expression between 12 tissues showed that the six *BnBRI1* genes clustered into one subclass and six *BnBRL* genes into another subclass, and the expression levels of *BnBRI1* genes were generally higher than the *BnBRL* genes (Figure 5B). Furthermore, Bn*BRI1.c*, Bn*BRI1.e*, and Bn*BRI1.f* had higher expression in various tissues compared to other *BnBRI1* genes. It is noteworthy *BnBRI1.f* gene was highly expressed in all tissues.

To generate new germplasm resources with a dwarf and optimized plant structure of *B. napus*, we designed single guide RNAs targeted to a common conserved region of *BnBRI1* genes (Figure 5C), inserted these sgRNA into the pHSE401 expression vector and transformed into hypocotyls of rapeseed cultivar K407 (Figure 5D). We obtained a total of 130 transgenic positive plants (Figure 5E). Interestingly, more than 34 T_0_ plants displayed retarded growth with dark green rolling leaves at the stage of seedling growth (Supplementary Figure 4), consistent with phenotypes of previously reported classical *bri1* mutants (Huang et al., 2021). About 42 plants (44%) exhibited an extreme dwarf phenotype (plant height 30–60 cm), and 45 plants (47%) showed dwarf phenotypes (61–80 cm), and 9 plants (9%) showed semi-dwarf phenotypes (81–120 cm) (Figure 5F).

### Reduced Expression of *BnBRI1* Homologs Leads to Semi-Dwarf Phenotypes in *Brassica napus*

**FIGURE 6:**
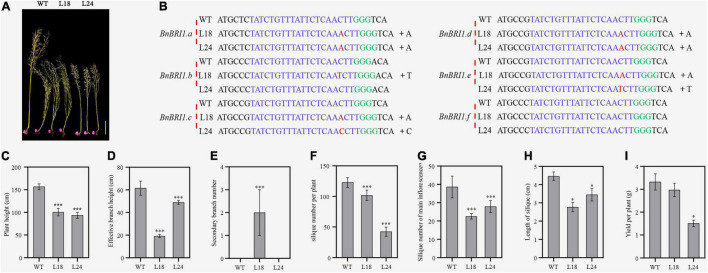
Sequencing results of *BnBri1* mutants and their agronomic traits. **(A)** Semi-dwarf material obtained by editing *BnBRI1* from left to right were WT (K407), L18 and L24 strains, scale bars, 20 cm. **(B)** Sequencing results of L18 and L24 strains. The sgRNA targeting sequences are highlighted in blue and the PAM sequences are green. **(C–I)** Statistical analysis of plant height, effective branch height, secondary branch number, silique number per plant, silique number of main inflorescence, yield per plant and length of silique in WT (K407), L18 and L24 homozygous plants from the greenhouse. Error bars standard deviation (*n* = 10). One-way analysis of variance was used for statistical analysis (“*,” corresponding to *P* < 0.05, and “^***^,” corresponding to *P* < 0.001).

To determine whether the transgenic lines had gene editing events, we selected the T_3_ generation plants of the semi-dwarf lines L18 and L24 for subsequent experiments (Figure 6A). The sequencing results of the single clones and PCR products near the sgRNA-targeted sites indicated that all target sites were edited in L24, except the *BnBRI1.b* and *BnBRI1.f* genes, similarly, all target sites were edited in L18, except the *BnBRI1.f* gene. The variations between the two lines were single base insertions of A, T or C (Figures 6B,C and Supplementary Figures 5, 6).

Furthermore, qRT-PCR was conducted (Supplementary Figure 7), and showed that the *BnBRI1* expression of the L18 and L24 were both reduced significantly. As a result, the hypocotyl length of both mutants shortened obviously (Supplementary Figures 8A,C). The results of the BRZ treatment showed that the hypocotyl length of the two mutants were significantly decreased under 1,000 nM, so the L18 and L24 were BRZ sensitive mutants (Supplementary Figures 8B,D). These verified that the both gene edited lines are indeed related to brassinostreoid signaling transduction pathway.

Agronomic traits were closely related to agricultural production. We planted the L18 and L24 lines in a greenhouse, and investigated the major agronomic traits, plant height, effective branch height, secondary branch number, silique number per plant, silique number of main inflorescence, length of silique and yield per plant (Figures 6C–I). We found that almost all traits exhibited significant differences between the control WT and the two gene edited lines. Specifically, compared with the control, L24 had fewer secondary branches, fewer silique number of main inflorescences, fewer siliques in the whole plant, and shorter siliques, resulting in a significantly lower yield than the control. In contrast, when the plant height and the branch length of L18 were significantly diminished compared to the control, the yield per plant was not reduced. Among yield-related traits, although L18 had shorter silique and a lower silique number of main inflorescence, the increased number of secondary branches increased the total number of silique and prevented a decline in yield.

## Discussion

LRR-RLKs sense extracellular signals and stimuli. In this study, we identified 444 *LRR-RLK* genes in the genome of ZS11, approximately twice as many as in *A. thaliana*. These 444 genes were further divided into 22 subfamilies, with LRR-RLK-III subfamily as the largest, whereas there were no genes in the I-2 subfamily. In general, a typical LRR-RLK consists of an extracellular tandem LRR domain, a transmembrane domain, and an intracellular protein kinase domain (Mishra et al., 2021), however, the I-1 subfamily also contains a malectin-like domain at the N-terminus. This domain is found in a different receptor-like kinase, known as the Catharanthus roseus receptor-like kinase 1-like proteins (CrRLK1Ls) (Franck et al., 2018). These findings highlight the domain similarity and functional complexity of *LRR-RLKs* family.

Gene duplication including tandem, tetraploid, segmental, and transpositional duplication, represents a major mechanism of new gene formation and gene family expansion (Freeling, 2009). Compared with the diploid *Arabidopsis*, many genes were significantly expanded in the allopolyploidization *Brassica napus*, likely due to genome polyploidy. Indeed, our research showed that 289 segmental duplications occurred across AA/CC subgenomes. Moreover, 51 and 55 duplication events occurred in AA and CC subgenomes, respectively. These repeated events within each subgenome may relate to their progenitors (*Brassica rapa* and *Brassica oleracea*). In addition, 6 tandem duplication events took place on chromosomes A04, A07, C04, C06, C07, and C09, respectively. Thus, at least three types of gene duplications appeared in the *BnLRR-RLKs* family: tetraploid, segmental and tandem duplication.

Gene expression patterns are closely related to their functions. We examined public RNA sequencing data to determine the expression patterns of *BnLRR-RLK* genes from 12 tissues in ZS11. We found that most *BnLRR-RLKs* have tissue-specific expression, for instance, *BnLRR-RLK-3C-12* and *BnLRR-RLK-3A-15* were highly expressed in stamens. Phylogenetic tree clustering suggested that both genes were clustered with a pollen tube related gene, *AtPRK4* (At3G20190). Based on these findings we speculate that the two highly expressed genes in stamens are related to pollen tube elongation and fertilization (Chang et al., 2013; Duckney et al., 2017). Similarly, we also found that *BnLRR-RLK-8A-12* and *BnLRR-RLK-8C-6* are highly expressed in the roots; its homologous gene *RLK7* (AT1G09970) in *Arabidopsis* is involved in the development of lateral roots, reflecting that the two genes are related to root morphogenesis (Toyokura et al., 2019).

Abiotic stress seriously compromises plant growth and development. We uncovered 132 *BnLRR-RLK*s from the expression matrix of various abiotic stresses, such as dehydration, NaCl, ABA, cold. We found that *BnLRR-RLK-8C-7* was highly expressed after 4 h cold treatment, whereas its homologous *SIF3* (AT1G51805), belongs to the *STRESS INDUCED FACTOR (SIF)* gene family, involved in plant basal defenses and pathogen resistance of *Arabidopsis* (Yuan et al., 2018). We propose that *BnLRR-RLK-8C-7* probably relate to the early cold stress response. *BnLRR-RLK-7A-5* was highly expressed only after a 4-h ABA treatment, and its homologous *AtBARK1* is involved in the BR signaling pathway, suggesting that this gene may play a role in ABA signaling pathway (Kim et al., 2013). Given the importance of *LRR-RLK* family members for plant growth, development, and stress tolerance, our study of tissue-specific and abiotic stress expression patterns facilitates the discovery of *LRR-RLK* genes with important biological functions and their underlying regulatory mechanisms.

BR signaling requires the *BRI1* and other *LRR-RLKs*. The six *BnBRI1* genes have similar motif elements and intron-exon structure, supporting their homology and evolutionary conservation. Nevertheless, the six *BnBRI1* have tissue-specific expression patterns: *BnBRI1.c, BnBRI1.e, BnBRI1.f* were highly expressed in various tissues; in particular, *BnBRI1.f* was highly expressed in all tested tissues. We propose that the six copies of *BnBRI1* have different functions despite their similarities.

Gene editing technology can be used to improve agronomic trait in crops and accelerate the breeding process. This technology was used in rapeseed to knockout the *BnaA03.BP* gene, which generated semi-dwarf plants without affecting other traits (Fan et al., 2021). Knockout of the two *BnaMAX1* homologous improved plant architecture and increased yield (Zheng et al., 2020). Editing the *BnD14* gene resulted in a compact architecture and is expected to achieve high-density plants in production (Stanic et al., 2021).

*BRI1* mutations caused developmental defects, including extreme dwarfism and male sterility (Clouse et al., 1996; Yamamuro et al., 2000; Feng et al., 2015; Huang et al., 2021). We used CRISPR/cas9 technology to edit the six *BnBRI1* genes in rapeseed, and obtained 130 gene-edited rapeseed plants, some of which exhibited a dwarf or semi-dwarf phenotype. Target identification in the two semi-dwarf strains revealed that all target genes were edited except *BnBRI1.b* and *BnBRI1.f* in L24, and *BnBRI1.f* in L18. Although the *BnBRI1.f* gene was not edited, we found one nucleotide variation in this gene compared to other five *BnBRI1* genes that causes a F689S amino acid change, which probably relates to the phenotypes of L18 and L24. Among yield-related traits, although L18 had shorter silique and decreased silique number of main inflorescence, the increased number of secondary branches led to more silique and ultimately prevented a decline in yield. As a semi-dwarf line with excellent agronomic characteristics, L18 could be used to construct an ideotype plant structure of rapeseed.

## Conclusion

This study systematically analyzed the *LRR-RLKs* family in *B. napus*. A total of 444 genes were detected in the genome of cultivar ZS11, which were divided into 22 groups based on phylogenetic relationships. These genes were widely and asymmetrically distributed across 19 chromosomes, and exhibited tissue- and stress- specific expression profiles. Moreover, we identified and knocked out *BnBRI1* homologs to generate semi-dwarf lines with increased yield. This study lays the foundation to investigate *LRR-RLK* family gene function, expands the “ideotype” germplasm resources for field breeding, and accelerates the process of rapeseed breeding.

## Data Availability Statement

The datasets presented in this study can be found in online repositories. The names of the repository/repositories and accession number(s) can be found in the article/Supplementary Material.

## Author Contributions

YFZ and JM designed the study. MS and BL performed the analysis and wrote the manuscript. RA, YTZ, FL, and SHH contributed to genetic transformation of *Brassica napus*. SWH and CX revised the manuscript. All authors have read and approved the final manuscript version.

## Conflict of Interest

The authors declare that the research was conducted in the absence of any commercial or financial relationships that could be construed as a potential conflict of interest.

## Publisher’s Note

All claims expressed in this article are solely those of the authors and do not necessarily represent those of their affiliated organizations, or those of the publisher, the editors and the reviewers. Any product that may be evaluated in this article, or claim that may be made by its manufacturer, is not guaranteed or endorsed by the publisher.

## Footnotes

1
https://www.arabidopsis.org/

2
https://ngdc.cncb.ac.cn/

3
http://pfam.xfam.org/

4
http://www.ncbi.nlm.nih.gov/Structure/cdd/wrpsb.cgi

5
http://smart.embl.de/

6
http://www.expasy.org/

## Supplementary Material

Supplementary Figure 1 Phylogenetic relationship, conserved motifs and gene structures of BnLRR-RLK proteins. **(A)** Phylogenetic relationship of *BnLRR-RLKs*. **(B)** Conserved motifs of BnLRR-RLKs, boxes of different colors represent different motifs. **(C)** Gene structure of BnLRR-RLKs. The green and yellow boxes represent the coding sequences (CDSs) and untranslated regions (UTRs), respectively, and black lines represent introns.

Supplementary Figure 2 Chromosomal localization of *BnLRR-RLK* genes across *B. napus* chromosomes. Words marked in red indicate tandem duplications. The location of each gene was determined based on its physical location on the chromosome.

Supplementary Figure 3 The synteny of *BnLRR-RLK* genes in genomes of *Arabidopsis* and *Brassica napus*. The light-gray lines represent all collinear blocks between *B. napus* and *A. thaliana*, and light-purple lines represent orthologous relationships between *BnLRR-RLKs* and *AtLRR-RLKs*.

Supplementary Figure 4 Phenotypes of CRISPR/Cas9 edited *BnBRI1* strong mutantats at seedling stage. The *bri1* strong mutants showed significant growth retardation with dark green and edge valgus thick leaves, and were eventually lost due to failure to harvest seeds, scale bars, 1.2 cm.

Supplementary Figure 5 Sequencing chromatograms of positive single clones in T_3_ generation. Each single clone was evaluated by Sanger sequencing. The inserted nucleotides are highlighted with yellow colors, pointing by black arrows.

Supplementary Figure 6 Sequencing chromatograms of PCR products in T_3_ generation. Each PCR product was purified and sequenced. The inserted nucleotides are highlighted by black box.

Supplementary Figure 7 Expression of *BnBRI1*genes in WT, L18 and L24. The *BnUBC21* gene was used as an internal control. Error bars represent SE (standard errors) of three independent replicates. Different lowercase letters indicate values significant differences compared to WT at the level *P* < 0.05.

Supplementary Figure 8 Hypocotyl length and response to BRZ in gene edited lines. **(A)** Phenotypes of the gene edited lines at seedling stage. **(B)** The gene edited lines under BRZ treatment. **(C)** Hypocotyl length of seedlings shown in **(A)**. Data are the mean ± *SD* (*n* = 15), scale bars, 1 cm. **(D)** Hypocotyl length of seedlings at different BRZ concentrations shown in **(B)**, scale bars, 1.5 cm. Different letters indicate significant differences based on one-way ANOVA (*P* < 0.05).
