# Supplementary figures and images for: Genome-Wide Survey of Leucine-Rich Repeat Receptor-Like Protein Kinase Genes and CRISPR/Cas9-Targeted Mutagenesis BnBRI1 in Brassica napus
Source: Front Plant Sci. 2022 Apr 12;13:865132. doi: 10.3389/fpls.2022.865132 (PMC9039726; doi:10.3389/fpls.2022.865132)

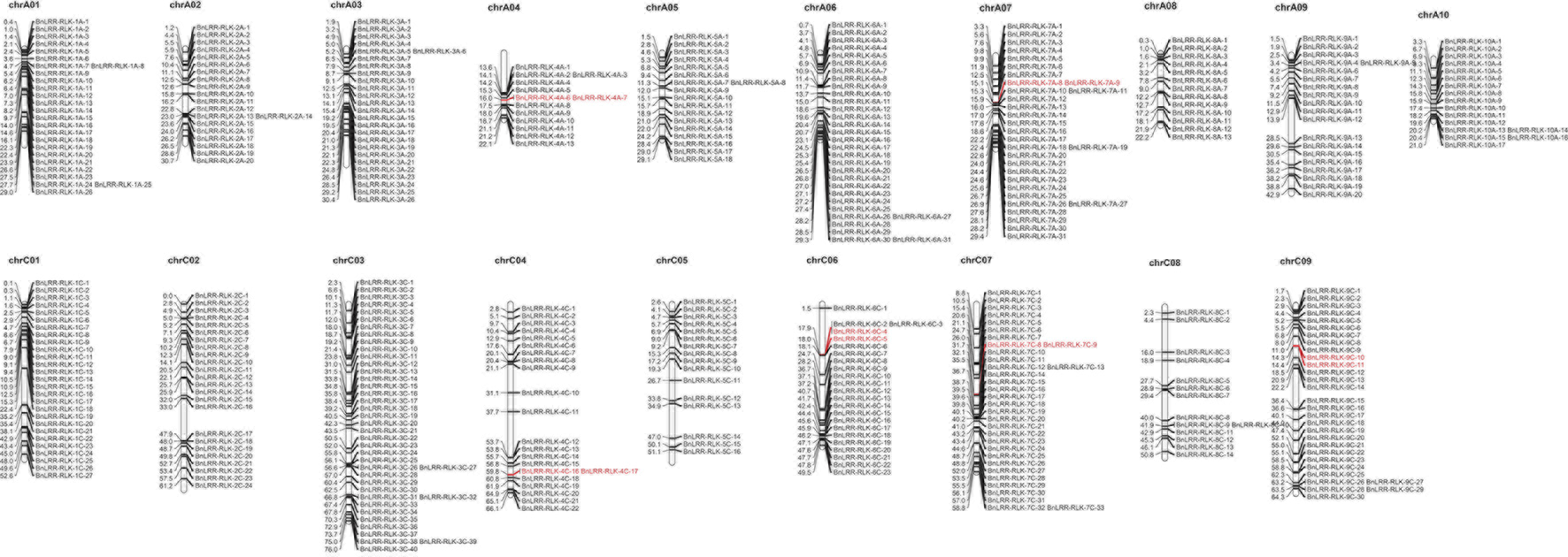

Supplement: Supplementary Figure 2 — Chromosomal localization of BnLRR-RLK genes across B. napus chromosomes. Words marked in red indicate tandem duplications. The location of each gene was determined based on its physical location on the chromosome. [file Image_2.tiff]

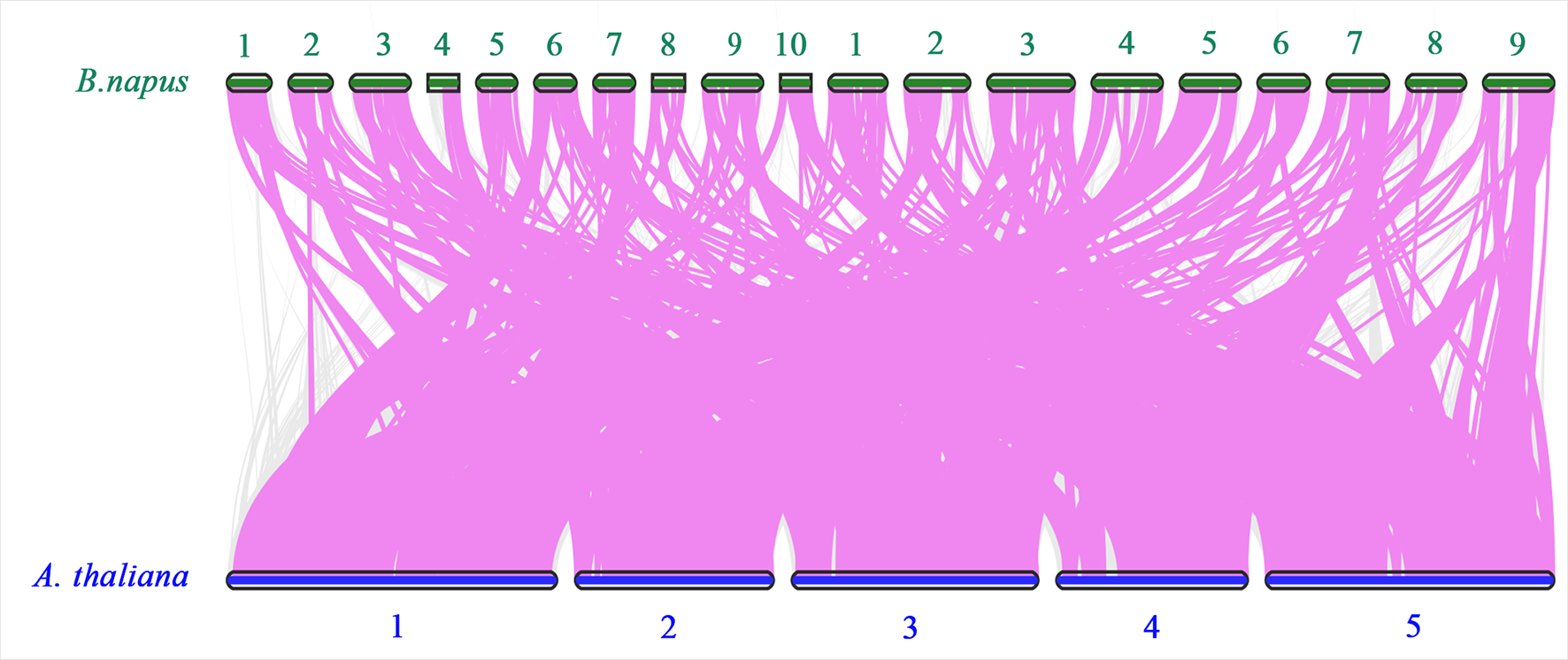

Supplement: Supplementary Figure 3 — The synteny of BnLRR-RLK genes in genomes of Arabidopsis and Brassica napus. The light-gray lines represent all collinear blocks between B. napus and A. thaliana, and light-purple lines represent orthologous relationships between BnLRR-RLKs and AtLRR-RLKs. [file Image_3.tif]

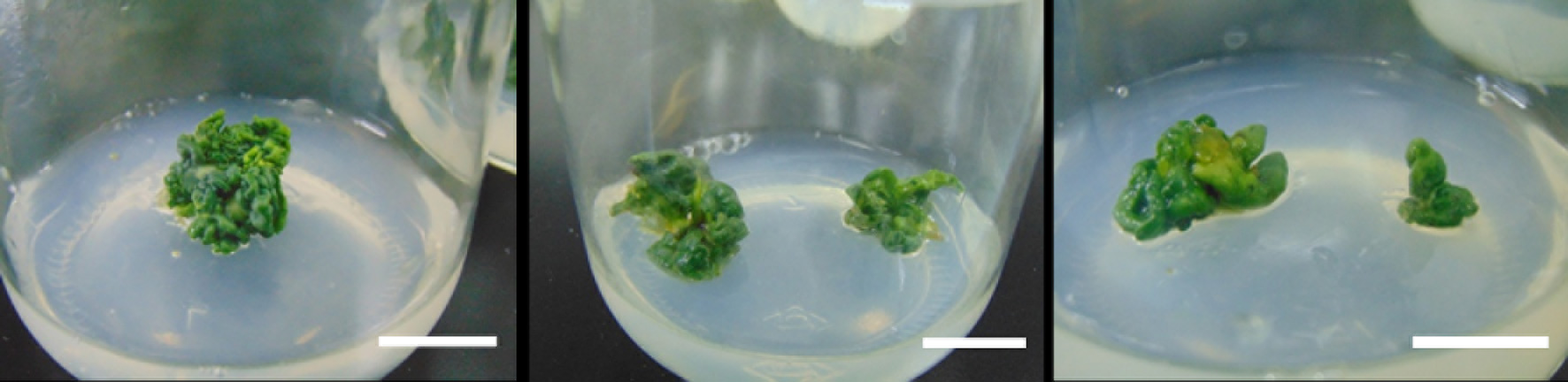

Supplement: Supplementary Figure 4 — Phenotypes of CRISPR/Cas9 edited BnBRI1 strong mutantats at seedling stage. The bri1 strong mutants showed significant growth retardation with dark green and edge valgus thick leaves, and were eventually lost due to failure to harvest seeds, scale bars, 1.2 cm. [file Image_4.tiff]

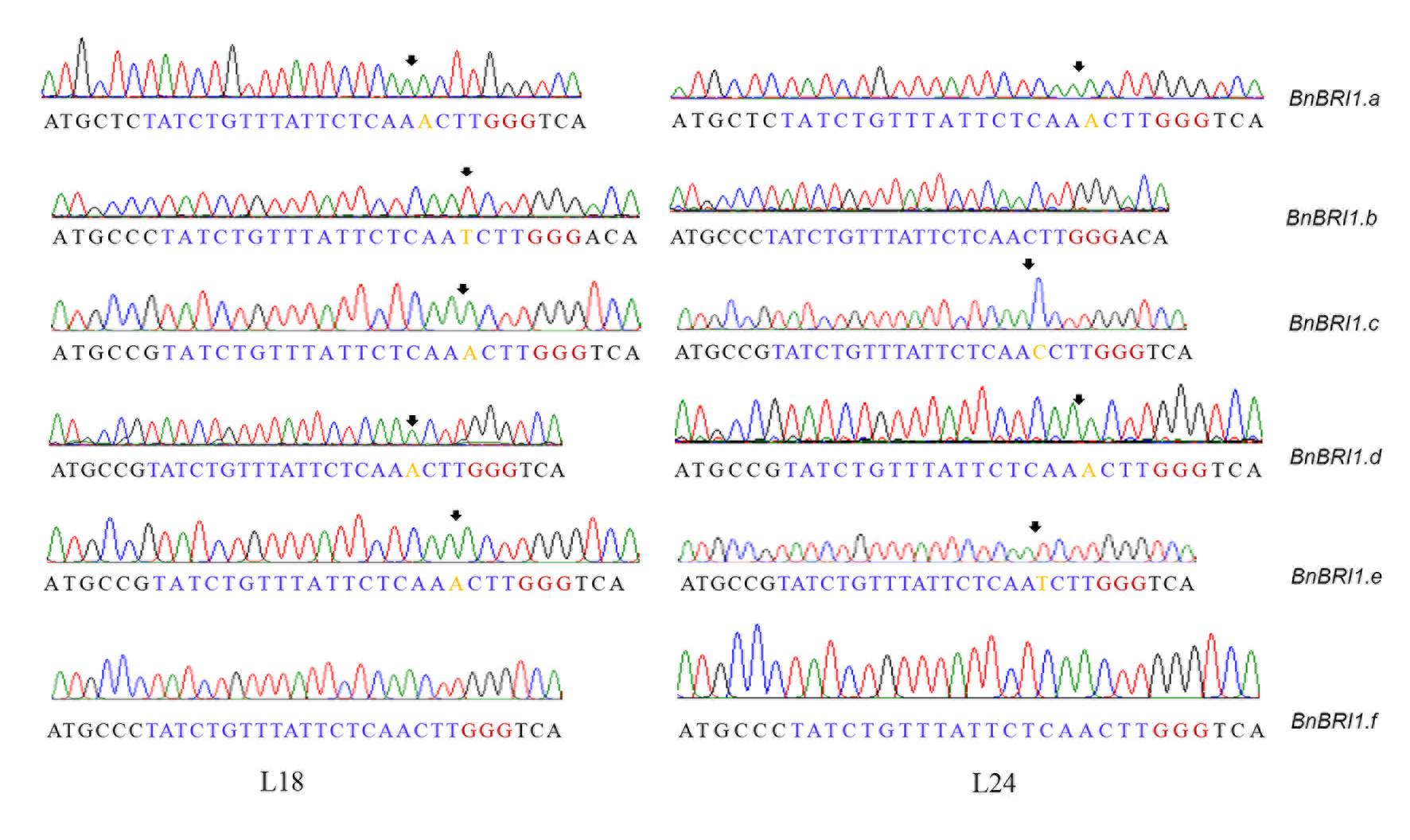

Supplement: Supplementary Figure 5 — Sequencing chromatograms of positive single clones in T3 generation. Each single clone was evaluated by Sanger sequencing. The inserted nucleotides are highlighted with yellow colors, pointing by black arrows. [file Image_5.tiff]

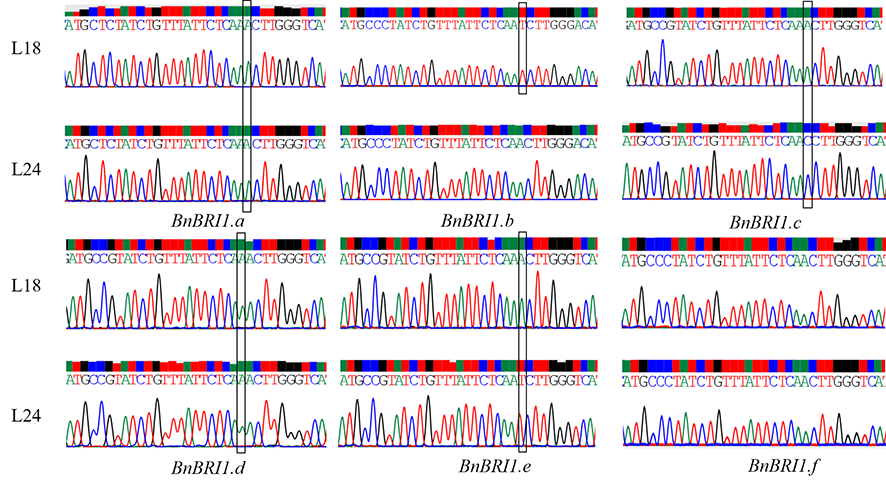

Supplement: Supplementary Figure 6 — Sequencing chromatograms of PCR products in T3 generation. Each PCR product was purified and sequenced. The inserted nucleotides are highlighted by black box. [file Image_6.tiff]

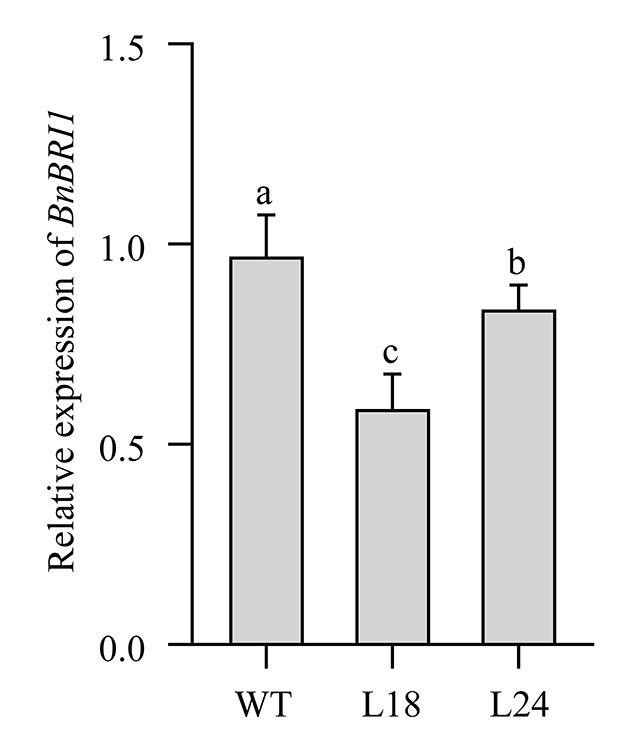

Supplement: Supplementary Figure 7 — Expression of BnBRI1genes in WT, L18 and L24. The BnUBC21 gene was used as an internal control. Error bars represent SE (standard errors) of three independent replicates. Different lowercase letters indicate values significant differences compared to WT at the level P < 0.05. [file Image_7.tiff]

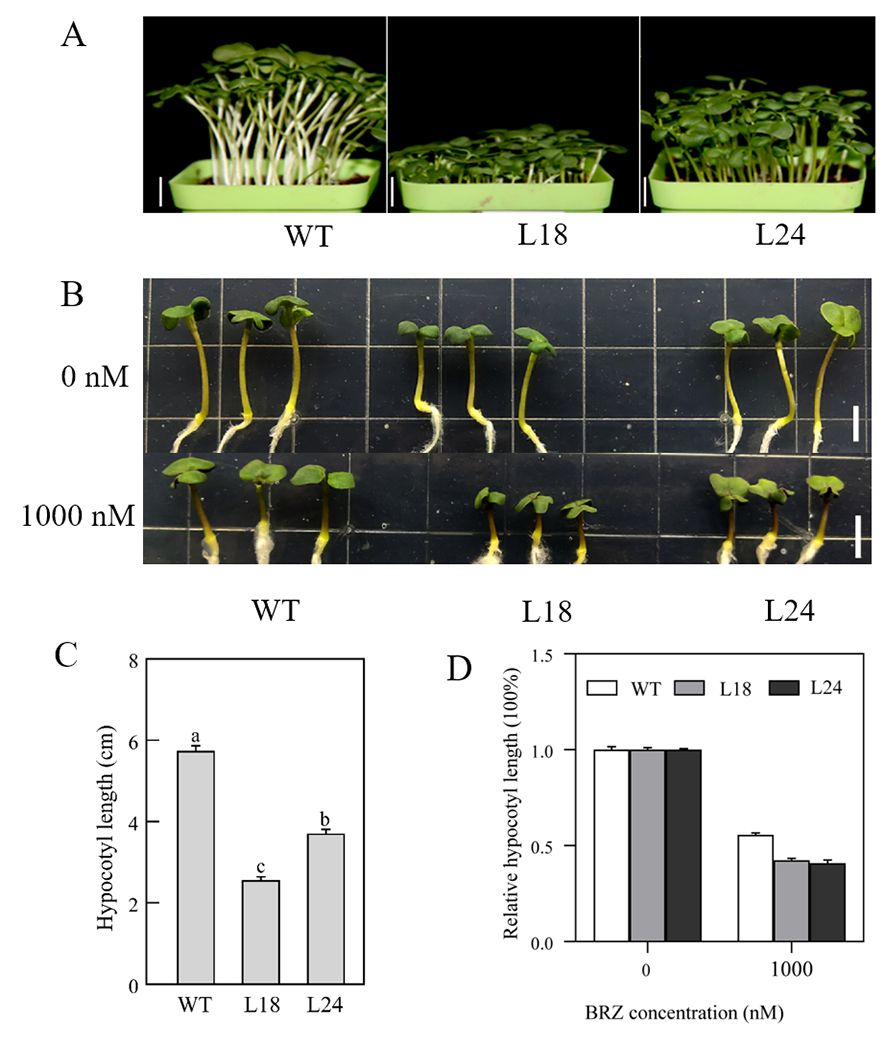

Supplement: Supplementary Figure 8 — Hypocotyl length and response to BRZ in gene edited lines. (A) Phenotypes of the gene edited lines at seedling stage. (B) The gene edited lines under BRZ treatment. (C) Hypocotyl length of seedlings shown in (A). Data are the mean ± SD (n = 15), scale bars, 1 cm. (D) Hypocotyl length of seedlings at different BRZ concentrations shown in (B), scale bars, 1.5 cm. Different letters indicate significant differences based on one-way ANOVA (P < 0.05). [file Image_8.tiff]
