# Supplementary material for: Genome-Wide Survey of Leucine-Rich Repeat Receptor-Like Protein Kinase Genes and CRISPR/Cas9-Targeted Mutagenesis BnBRI1 in Brassica napus
Source: Front Plant Sci. 2022 Apr 12;13:865132. doi: 10.3389/fpls.2022.865132 (PMC9039726; doi:10.3389/fpls.2022.865132)
Supplement: Supplementary Figure 1 — Phylogenetic relationship, conserved motifs and gene structures of BnLRR-RLK proteins. (A) Phylogenetic relationship of BnLRR-RLKs. (B) Conserved motifs of BnLRR-RLKs, boxes of different colors represent different motifs. (C) Gene structure of BnLRR-RLKs. The green and yellow boxes represent the coding sequences (CDSs) and untranslated regions (UTRs), respectively, and black lines represent introns. [file Image_1.pdf]

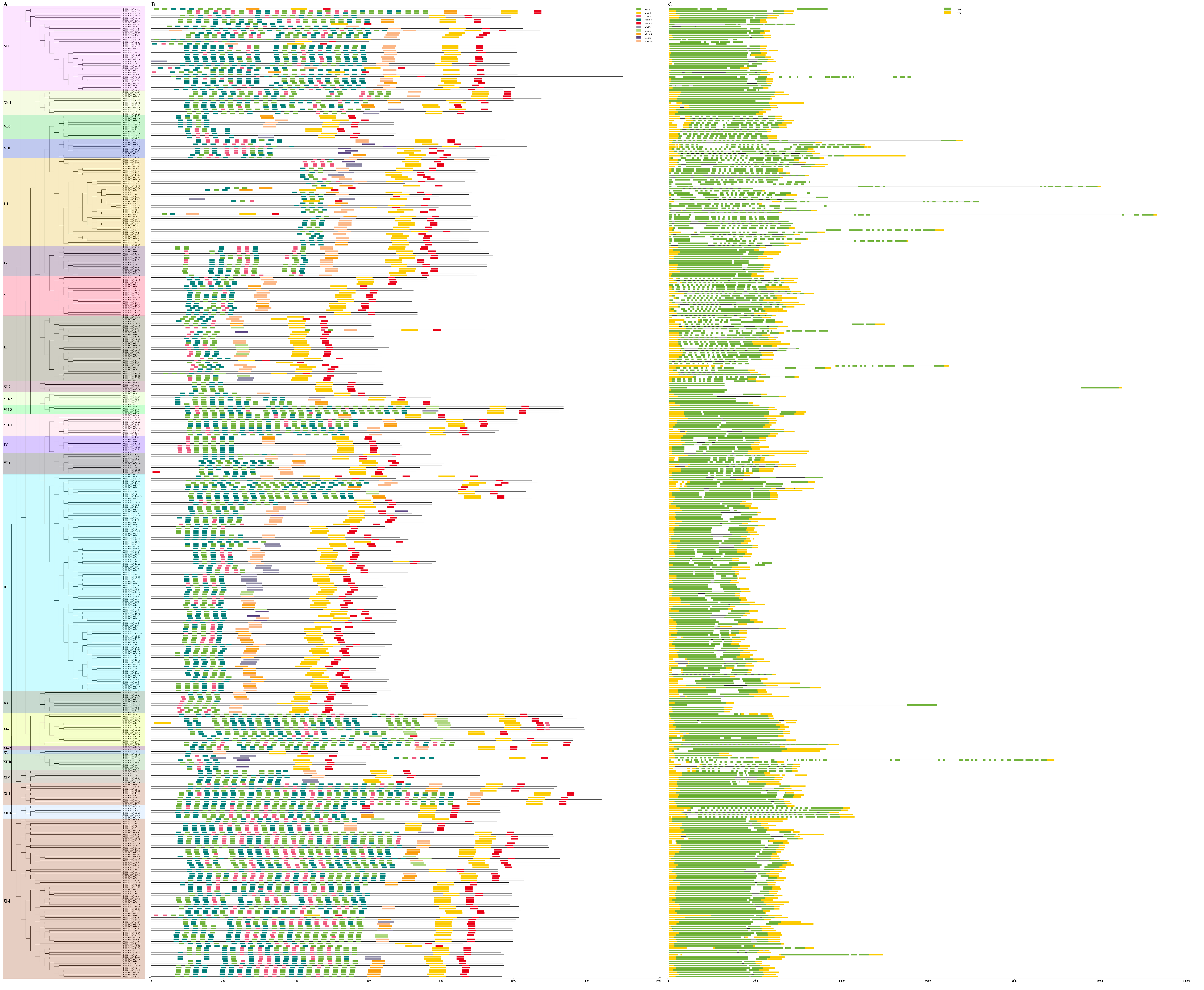
A
B
C
XII
Xb-1
VI-2
VIII
I-1
IX
V
II
XI-2
VII-2
VII-3
VII-1
IV
VI-1
III
Xa
Xb-1
Xb-2
XV
XIIIa
XIV
XI-1
XIIIb
XI-1
Motif 1
Motif 2
Motif 3
Motif 4
Motif 5
Motif 6
Motif 7
Motif 8
Motif 9
Motif 10
CDS
UTR
0
200
400
600
800
1000
1200
1400
0
3000
6000
9000
12000
15000
18000
